# Chylous Ascites Related to Radiotherapy in a Patient With Gastric Cancer

**DOI:** 10.7759/cureus.38716

**Published:** 2023-05-08

**Authors:** Leidy Barrera, Diego Gonzalez, Luisa Pelaez

**Affiliations:** 1 General Medicine, Universidad Pontificia Bolivariana, Medellin, COL; 2 Oncology, Instituto de Cancerologia Las Américas, Medellin, COL; 3 Oncology Nursing, Instituto de Cancerologia Las Américas, Medellin, COL

**Keywords:** lymphangiography, radiotherapy complications, gastric adenocarcinoma, chylous ascites, ascites

## Abstract

Chylous ascites is an uncommon complication seen in patients who have received radiotherapy to the abdomen. However, the morbidity caused by peritoneal ascites makes this an important complication to be considered when giving abdominal radiation to oncology patients*.* We present the case of a 58-year-old woman with gastric adenocarcinoma, who consulted for recurrent ascites after receiving abdominal radiotherapy as an adjuvant treatment to surgery. Different tests were carried out to evaluate the cause. Malignant abdominal relapse and infection were ruled out. Paracentesis evidenced swallowed fluid, therefore, the possibility of chylous ascites due to radiotherapy was considered. Intrathoracic, abdominal, and pelvic lymphangiography was performed with Lipiodol and the absence of cisterna chyli was confirmed as the cause of refractory ascites. After the diagnosis, the patient went under aggressive in-hospital nutritional support treatment, with clinico-radiological response.

## Introduction

Gastric cancer is one of the main causes of morbidity and mortality in the world [1]. In Colombia, this is the main cause of death due to cancer [2], which is explained in part by a late diagnosis, related in many cases to cancer in advanced stages [3]. Delayed diagnosis decreases cure rates and increases the risk of regional and distant relapse.

Multimodal management (surgery, chemotherapy, radiotherapy) is used to treat this kind of neoplasia. Nowadays, there are some protocols such as Adjuvant Chemoradiation Therapy in Stomach Cancer (ARTIST) and ARTIST 2, adjuvant capecitabine plus oxaliplatin for gastric cancer after D2 gastrectomy (CLASSIC), and adjuvant chemotherapy with fluorouracil, leucovorin, oxaliplatin, docetaxel (FLOT), which have significantly improved patient survival. However, it is important to remember that some of those treatments are linked to side effects and therefore morbidity. Health caregivers have the complex task of recognizing the clinical settings related to the relapse of the illness and the differential diagnosis, which sometimes includes complications related to the treatments. 

## Case presentation

A 58-year-old female was diagnosed with cT2N2M0 gastric adenocarcinoma and treated with subtotal gastrectomy + lymph node dissection on July 25, 2018, followed by adjuvant treatment with ARTIST protocol, which consisted of three cycles of capecitabine and cisplatin (XP), followed by chemoradiotherapy with only capecitabine and then three additional cycles of XP. The radiotherapy phase was from November 22, 2018, to December 28, 2018, with a total dose of 45Gy administered in 25 fractions of 1.8Gy, and chemotherapy ended on April 12, 2019.

**Figure 1:**
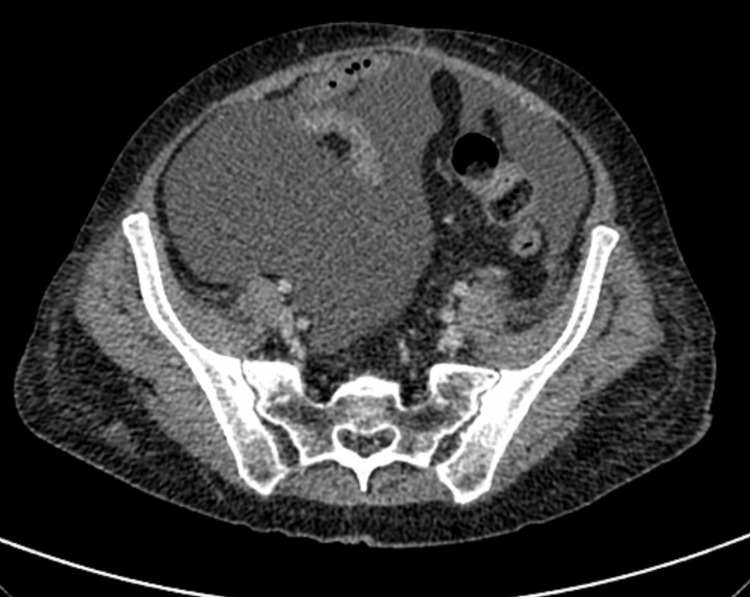
CT scan of abdomen, axial plane: free peritoneal fluid.

In June 2019, she complained of diarrhea, abdominal pain, and weight loss. She received treatment for actinic enteritis with little response and later developed ascites and lower extremity edema. Cancer relapse was suspected and images were taken. Computed tomography showed a large amount of free peritoneal fluid and nodular thickening (Figure 1).

The patient was taken for laparoscopy and peritoneum biopsy, which did not show any peritoneal implants, and pathology was negative for malignancy. The Ziehl-Neelsen and methenamine silver stains were also negative. The study of the peritoneal liquid showed the presence of exudate, triglycerides level +500mg/dl, and a serum-ascites albumin gradient of 0.7g/dl, which ruled out the presence of portal hypertension.

**Figure 2:**
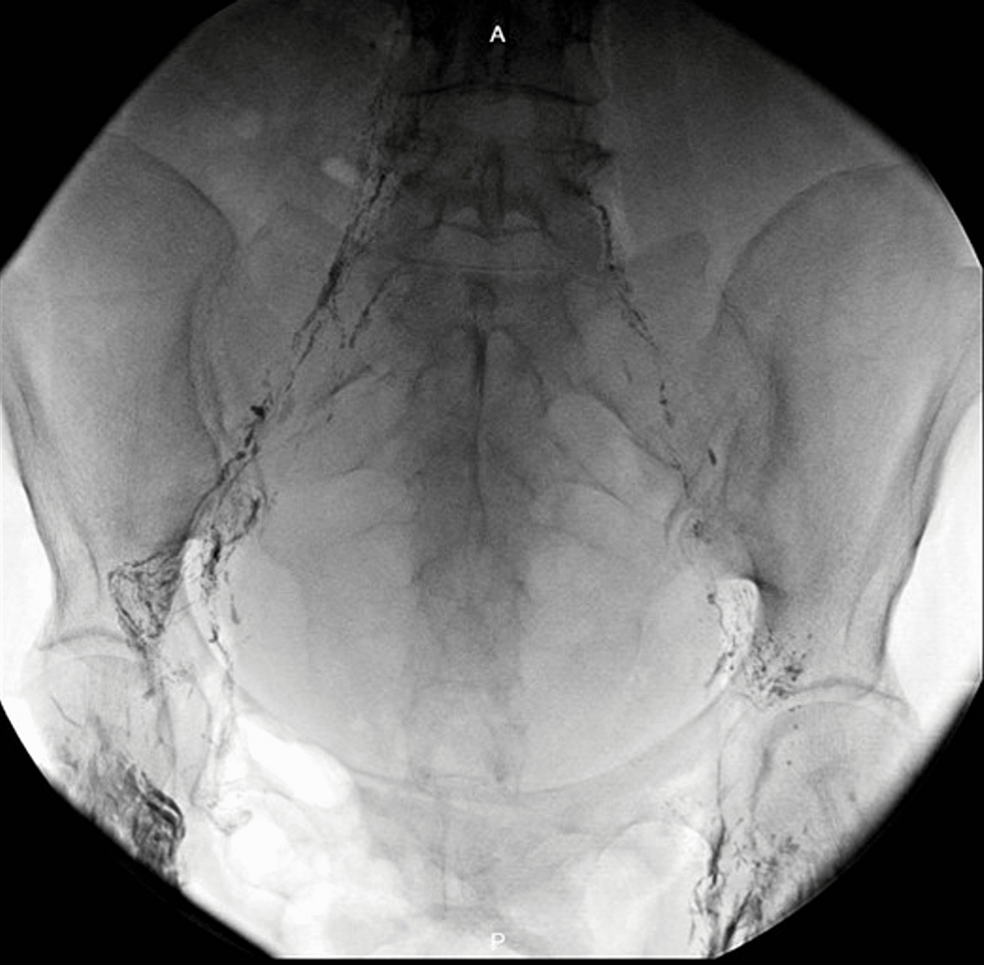
Lipiodol lymphangiography. Injected Lipiodol flowed up, with slow progression through the left lower limb. No extravasation of lipiodol was observed.

**Figure 3:**
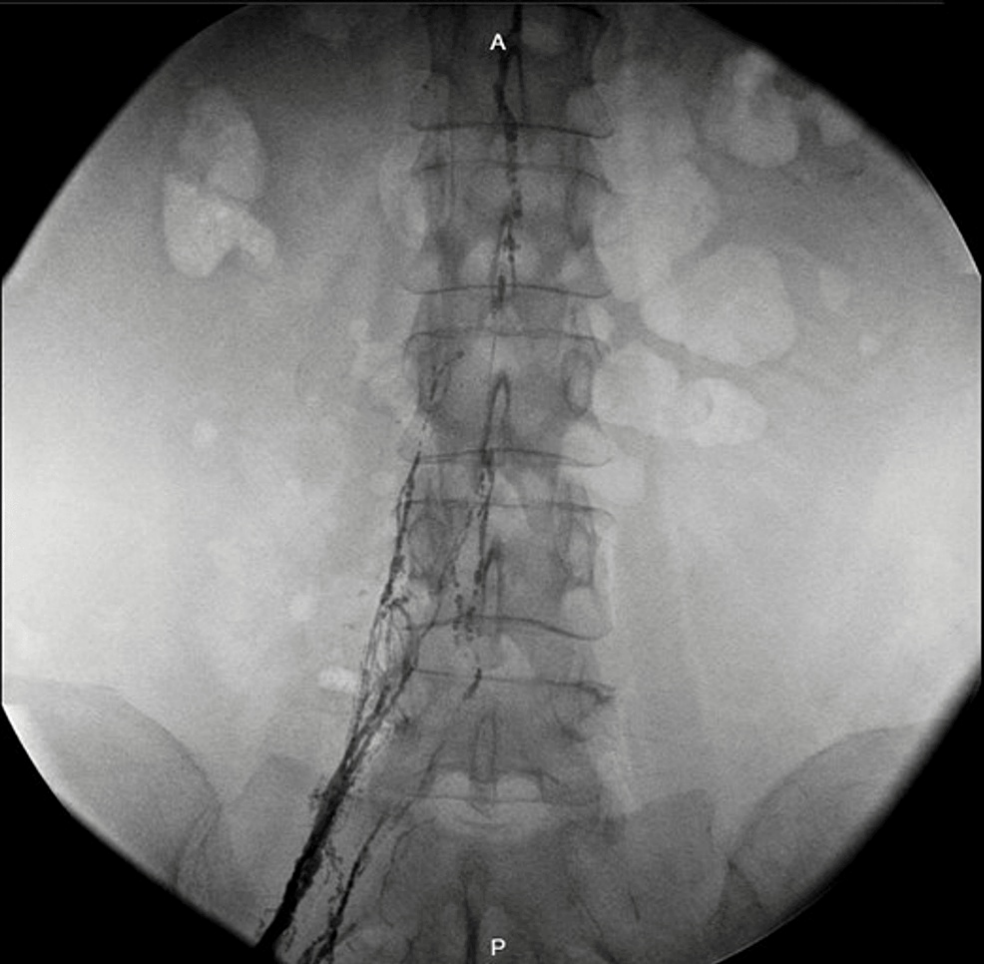
Lipiodol lymphangiography. Absence of the cisterna chyle.

The patient underwent paracentesis repeatedly and had a loss of 10kg in three months. She had three cytology results (cytospin and cell block method), all negative for malignancy. In December 2019, the paracentesis result depicted swallowed fluid, therefore, after taking into account this and the previous laboratory results, the possibility of chylous ascites due to radiotherapy was considered. Intrathoracic, abdominal, and pelvic lymphangiography was performed with Lipiodol (Figures 2, 3).

Given the suspicion of refractory chylous ascites secondary to oncological treatment (radiotherapy), and the severe physical involvement with a loss of 15kg by then, aggressive in-hospital nutritional support treatment was started. The patient was in a high-level complexity hospital for three weeks under a treatment based on mixed nutrition: parenteral nutrition plus enteral oral intake. The Intestinal Recovery Group prescribed a parenteral plan that started on March 6, 2020, with 607 kcal (15 kcal/kg with 0.6g/kg protein and 0.3g/kg lipids) for a coverage of 36% of the caloric goal. The composition was increased gradually until reaching a parenteral infusion with 1769 kcal on March 13, 2020 (30 kcal/kg plus 500 kcal due to malnutrition, 1.5g/kg protein, and 1.2g/kg lipids) achieving a 99% caloric coverage goal.

After 10 days of intensive treatment, the patient had gained 3kg, and her ascites had diminished; therefore, the Group decided to start cyclic parenteral nutrition, which was well tolerated. One week later, she was discharged with a home parenteral nutrition program based on a 12-hour nocturnal infusion. Additional to nutritional treatment, the patient received furosemide, spironolactone, and folic acid.

**Figure 4:**
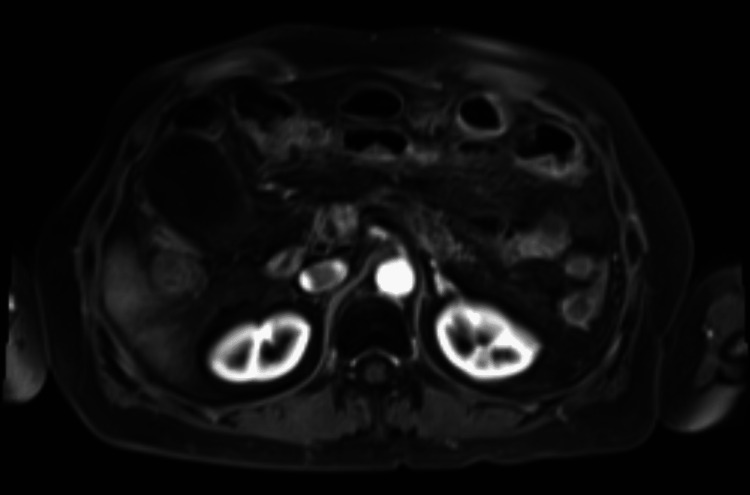
MRI, axial plane: small amount of free peritoneal fluid

She had ambulatory follow-up by the Recovery Intestinal Group for five months and the nutrition was finally suspended in August 2020. In October 2020, the patient was followed up by oncology, showing both clinical (no ascites, weight of 56kg, albumin 3.5gr/dl) and imaging responses (Figure 4). There was no availability of a Denver catheter to try to create a peritoneum-venous fistula. However, the patient responded to the supportive treatment. Oncology follow-up continued every three months, the latest assessment was in October 2022 with no evidence of cancer relapse. In addition, laboratory results showed albumin in the normal range (4.37gr/dl) and the patient had gained 15kg since the nutritional treatment.

## Discussion

Peritoneal involvement manifested as ascites is one of the main findings related to the relapse of gastric cancer [4]. Although the causes of ascites are multiple [5], the initial approach rarely involves the search for alternative scenarios to relapse, since, in general terms, patients have previous diagnoses of gastric cancer in advanced stages, and the pre-test probability of peritoneal carcinomatosis is very high such that peritoneal biopsy is not necessary.

However, there are some cases in which the clinical course makes it necessary to perform additional studies to confirm the cause of ascites. In this patient, recurrent ascites with chylous fluid, associated with a history of intra-abdominal radiotherapy, generated the suspicion of a non-malignant cause of ascites [6]. The need for a histological diagnosis was insisted on; laparoscopy ruled out macroscopic peritoneal involvement, and biopsies were also negative for malignancy and infection. All of the above raised the suspicion of chylous ascites secondary to radiotherapy toxicity, and the results of cytology, cultures, and images made it possible to rule out some of the differential diagnoses of chylous ascites such as malignancy, tuberculosis, and traumatic obstruction.

Chylous ascites associated with radiotherapy is a complication rarely described in the literature [7-8]. Lymphoscintigraphy and lymphangiography are required for diagnosis, and in the case of the patient, the imaging study showed the absence of cisterna chyle, which is considered a sequel to radiotherapy and explains the clinical picture. Besides the diagnostic role of identifying lymphatic leaks, some reports have also suggested a therapeutic role in cases with lymphatic leakage. Medications such as orlistat, somatostatin analogs, and etilefrine have been used with variable results [9-10].

On the other hand, it is interesting that low-dose radiotherapy such as 8Gy administered in eight fractions, and even 4Gy administered in two fractions can be used to treat other causes of chylous ascites due to its sclerosing effect [11-12].

The management of this complication is based on nutritional measures and the use of nutritional supplements with a high-protein and low-fat diet with medium-chain triglycerides is recommended, but as malnutrition deepens, it becomes difficult to compensate for enteral requirements, since ascites impairs intestinal absorption, perpetuating the ascites/malnutrition cycle [10]. Therefore, interaction with specialized centers in advanced nutritional support is necessary, and the use of prolonged parenteral nutrition with all the inherent complications, as reported in the clinical case. 

Peritoneal-vascular shunts with the use of endovascular devices, such as the Denver type [13], are a valuable additional therapeutic alternative, but unfortunately, it was not available to our patient since it is not marketed in Colombia. Surgical shunts involve high surgical risk and vascular complications due to malnutrition. Currently, the patient is free of relapse of the disease and has adequate nutritional support.

## Conclusions

Although an infrequent complication, chylous ascites should be considered as a possible cause of refractory ascites in cancer patients who have received abdominal radiotherapy. A quick diagnosis could improve patients´ quality of life and a multimodal treatment that includes interventional radiology and intensive nutritional support could lead to a better result.
